# The Risk of Erectile Dysfunction in Chronic Obstructive Pulmonary Disease: A Population-Based Cohort Study in Taiwan

**DOI:** 10.1097/MD.0000000000000448

**Published:** 2015-04-10

**Authors:** Te-Chun Shen, Wen-Chi Chen, Cheng-Li Lin, Chia-Hung Chen, Chih-Yen Tu, Te-Chun Hsia, Chuen-Ming Shih, Wu-Huei Hsu, Fung-Chang Sung

**Affiliations:** From the Graduate Institute of Clinical Medicine Science (T-CS, C-HC, F-CS), College of Medicine; Division of Pulmonary and Critical Care Medicine (T-CS, C-HC, C-YT, T-CH, C-MS, W-HH), Department of Internal Medicine, China Medical University Hospital, Taichung; Division of Pulmonary and Critical Care Medicine (T-CS), Department of Internal Medicine, Chu Shang Show Chwan Hospital, Nantou; Department of Urology (W-CC); Management Office for Health Data (C-LL), China Medical University Hospital, Taichung, Taiwan.

## Abstract

The prevalence of erectile dysfunction (ED) in patients with chronic obstructive pulmonary disease (COPD) seemed high; however, large scale of population-based study was absent.

We conducted a retrospective cohort study using data from the National Health Insurance system of Taiwan. The cohort included 29,042 male patients who were newly diagnosed with COPD. Patients were recruited between 2000 and 2011, and the date of diagnosis was defined as the index date. Each patient was randomly matched with 1 male person from the general population without COPD according to age and the index year. The occurrence of ED was followed up until the end of 2011. The hazard ratios of ED were estimated using the Cox proportional hazard model after adjusting for age, index year, comorbidities, and medications.

The overall incidence of ED was 1.88-fold greater in the COPD cohort than in the non-COPD cohort (24.9 vs 13.3/1000 person-years, 95% confidence interval [CI] = 1.61–2.18). Compared with non-COPD patients, the hazard ratio increased with the number of emergency room visits and admissions for COPD from 1.51 (95% CI 1.29–1.77) to 5.46 (95% CI 3.03–9.84) and from 1.50 (95% CI 1.28–1.76) to 11.5 (95% CI 5.83–22.6), respectively.

Patients with COPD are at a significantly higher risk of developing ED compared with the general population regardless of age and presence of comorbidity. The results also support that poor control of COPD status is a key factor affecting ED development.

## INTRODUCTION

Chronic obstructive pulmonary disease (COPD) is a chronic, treatable, and preventable disease characterized by persistent airflow limitation caused by the long-term exposure to harmful particles or gases. Cigarette smoking is thought to be the greatest risk factor for the development of this disease. Increased inflammatory response in the airway is a typical feature in patients with COPD, although inflammation is not limited to the lungs and has systemic impact.^1^ Comorbidities such as cardiovascular diseases, lung cancer, osteoporosis, diabetes and metabolic syndromes, anxiety, depression, and various infections have all been associated with COPD.^2^

Erectile dysfunction (ED) is a condition of inability to persistently reach and/or maintain an erection sufficient to have satisfactory sexual activity.^3^ Aging, vascular insufficiency, psychogenic and neural disorders, systemic illness such as diabetes mellitus, hormonal derangement, and side-effects of medications may result in ED.^4^ ED has a profound negative impact on the quality of life and men's self-esteem. But, clinical practices may often underestimate this important problem.

Several studies have reported ED as a common comorbidity in patients with COPD. Fletcher and Martin^5^ first reported erectile impotence in 30% (6/20) of COPD patients. Köseoğlu et al^6^ reported a much higher ED prevalence of 75.5% (40/53) in COPD patients with various degrees of ED. The observed moderate-to-severe ED is 2.8-fold more prevalent in COPD patients than in controls (57% [54/95] vs 20% [6/30]).^7^ Collins et al also reported that 74% (67/90) of patients with moderate-to-severe COPD had at least 1 sexual dysfunction, with ED being the most common (72%, 48/67).^8^ A recent Turkey study has also confirmed lower testosterone levels in COPD patients with ED than non-COPD subjects with ED.^9^

Nevertheless, most of previous studies were performed either as a passive questionnaire or with small study samples. The present study attempts to determine the risk of ED in patients with COPD by conducting a nationwide population-based retrospective cohort study in Taiwan with data using the National Health Insurance Research Database (NHIRD) of Taiwan.

## MATERIALS AND METHODS

### Data Sources

In March 1995, the Taiwanese government officially started a National Health Insurance (NHI) program to provide a comprehensive, unified, and universal health insurance to all citizens of Taiwan (http://www.nhi.gov.tw/english/index.aspx). The NHIRD is a nationwide database of reimbursement claim data of the NHI program, and is maintained by the National Health Research Institutes (NHRI). We obtained the NHRI a subdataset of the Longitudinal Health Insurance Database 2000 (LHID 2000), which comprises a random sample of 1 million subjects with longitudinally linked data available from 1996 to 2011. The NHRI states that no statistical differences in age, sex, and health care costs exist between LHID 2000 and NHIRD. The NHRI has encrypted all patient identification numbers for the protection of privacy and provides researchers with anonymous numbers to link the relevant claim information, such as patient sex, birth date, medical claims, and types of care, including medication prescriptions. Diseases in the claims data were coded using the International Classification of Diseases, Ninth Revision, Clinical Modification (ICD-9-CM). An ad hoc committee was established for the insurance system to randomly sample claims to verify the diagnoses and related cares for accuracy to prevent violations. This study was approved by the Institutional Review Board of China Medical University in central Taiwan (CMU-REC-101-012).

### Study Population

Male patients with newly diagnosed COPD (ICD-9-CM 491, 492, and 496) were identified for the period of 2000 to 2011 from the dataset of LHID 2000. Subjects who had at least 2 diagnoses of COPD within a year with medication were eligible for inclusion in the COPD cohort. The first diagnosis date was defined as the index date of COPD. COPD patients with an ED history before the index date, aged <20 years, and with incomplete information on demographics were excluded. Comparison patients were selected from people without COPD or an ED history in the file of LHID 2000. For each identified COPD patient, 1 comparison person was randomly identified and frequency-matched with age (each 5-year span) and year of index date for the non-COPD cohort. Subjects <20 years of age were excluded.

### Outcome Measurements, Comorbidities and Medications

All study subjects were followed from the index date to until the date with ED diagnosed (ICD-9-CM code 302.72 and 607.84),^10^ date of withdrawal from the NHI program, or the end of 2011, whichever was reached first. The baseline comorbidities considered in this study included coronary artery disease (CAD) (ICD-9-CM code 410–414), peripheral artery disease (PAD) (ICD-9-CM code 443.81, 443.9, 440.2, 444.2, and 444.89), asthma (ICD-9-CM 493), stroke (ICD-9-CM code 430–438), kidney disease (KD) (ICD-9-CM code 580–589), hypertension (ICD-9-CM code 401–405), diabetes (ICD-9-CM code 250), hyperlipidemia (ICD-9-CM code 272), depression (ICD-9-CM code 296.2, 296.3, 300.4, and 311), and anxiety (ICD-9-CM code 300.00). Medications that may be associated with ED were also evaluated, including antihypertensive agents (anti-HTNs), benzodiazepines (BZDs), and nonsteroidal anti-inflammatory drugs (NSAIDs). Anti-HTNs included calcium channel blockers, angiotensin-converting enzyme inhibitors, angiotensin II receptor blockers, α-blocker, β-blocker, and diuretics.

### Statistical Analysis

The baseline characteristics and comorbidities of the COPD cohort and non-COPD cohort were compared. Chi-square and *t* tests were used to test the difference of categorical and continuous variables, respectively, between the 2 cohorts. The overall, sex-, age-, and comorbidity-specific incidence rates (per 10,000 person-years) of ED were calculated for each cohort. Univariable and multivariable Cox proportional hazards regression analyses were used to assess the hazard ratio (HR) and 95% confidence interval (CI) of ED development associated with COPD, compared with the non-COPD cohort. In the multivariable analysis, the model was adjusted for age and comorbidities of CAD, PAD, asthma, stroke, KD, hypertension, diabetes, hyperlipidemia, depression, anxiety, medications of anti-HTNs, BZDs, and NSAIDs, and all of which showed a significant difference (Table 1). The joint effect for ED between COPD and comorbidity was also assessed. The relationship between ED and the annual number of visits to the emergency room and admissions for COPD were also assessed. The cumulative incidence of ED between the COPD cohort and the non-COPD cohort was analyzed using the Kaplan–Meier method, and the difference was examined by log-rank test. The SAS software (version 9.2 for Windows; SAS Institute Inc, Cary, NC) was used for all data analyses. A *P* value <0.05 was considered statistically significant.

**TABLE 1:**
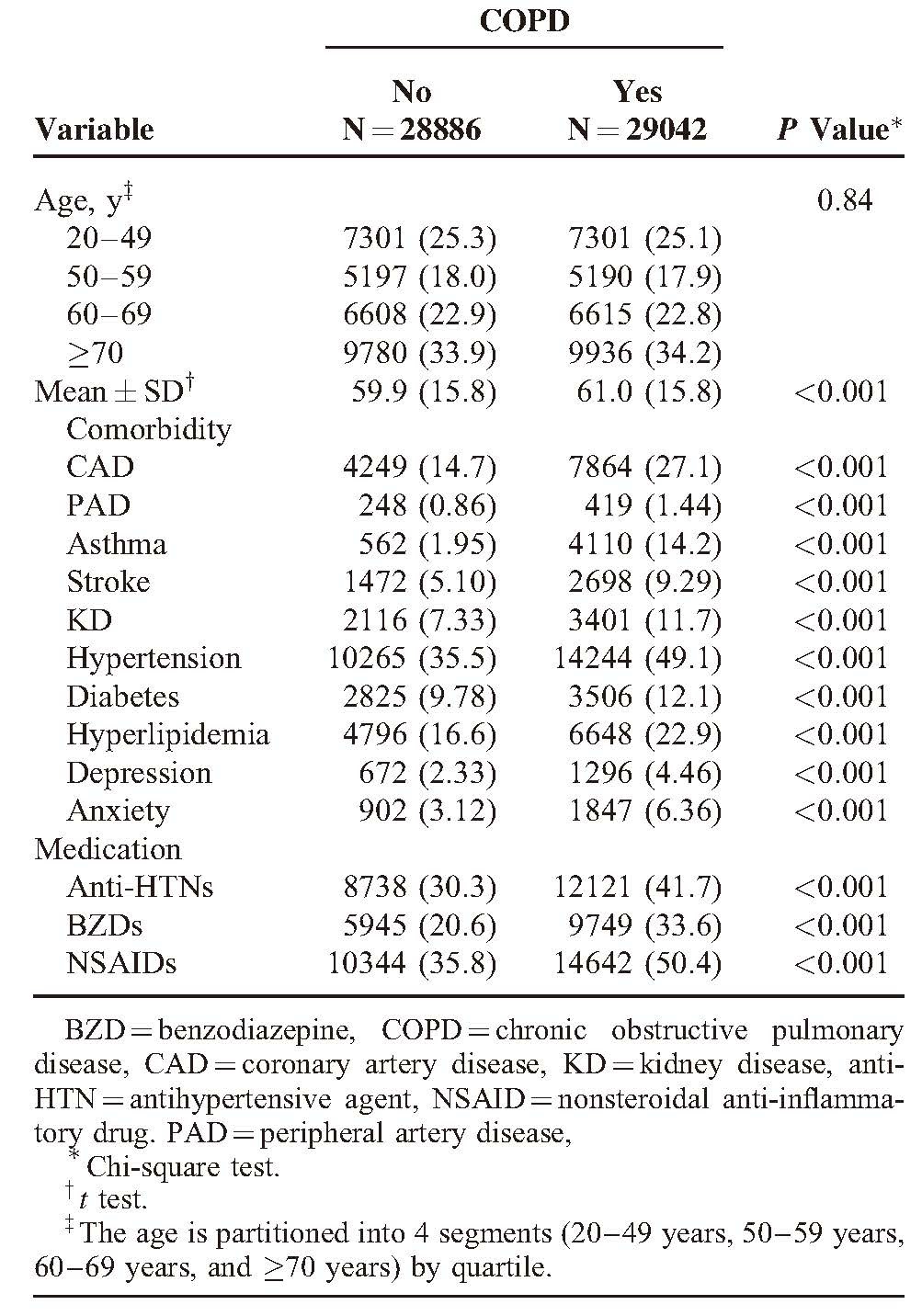
Comparisons in Demographic Characteristics and Comorbidities Between Cohorts With and Without COPD

## RESULTS

**FIGURE 1:**
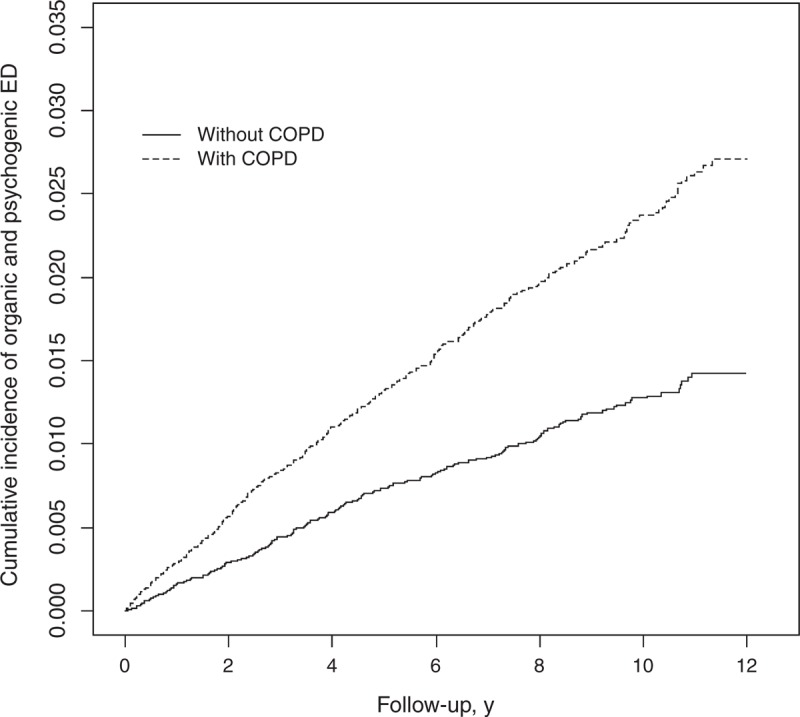
Cumulative incidence of ED in patients with (dashed line) or without (solid line) COPD. COPD = chronic obstructive pulmonary disease, ED = erectile dysfunction.

Overall, 57,928 subjects were selected for this retrospective cohort study, including 29,042 COPD patients and 28,886 non-COPD controls. The age distribution was similar in both cohorts, with the mean age slightly higher in the COPD cohort than in the non-COPD cohort (61.0 [SD = 15.8] vs 59.9 [SD = 15.8] years) but statistically significant. Compared with non-COPD subjects, COPD patients were more prevalent with comorbidities, including CAD, PAD, asthma, stroke, KD, hypertension, diabetes, hyperlipidemia, depression, and anxiety (all *P* < 0.001, Table 1). All of medications were more prevalent in the COPD cohort at the baseline (all *P* < 0.001, Table 1), compared with the non-COPD cohort. After 12 years of follow-up, the cumulative incidence of ED in the COPD cohort was approximately 1.29% higher than that in the non-COPD cohort (*P* < 0.001, Figure 1).

Overall, COPD patients had a 1.88-fold higher incidence of ED than non-COPD patients had (24.9 vs 13.3/1000 person-years, 95% CI 1.61–2.18) (Table 2). After adjusting for age and comorbidities of CAD, PAD, asthma, stroke, KD, hypertension, diabetes, hyperlipidemia, depression, and anxiety, COPD patients had an adjusted HR of 1.52 (95% CI 1.30–1.79) for ED, compared with non-COPD patients. COPD patients had approximately similar hazards to develop organic and psychosexual EDs. The association between psychosexual ED and COPD was not significant, with a small number of patients.

**TABLE 2:**
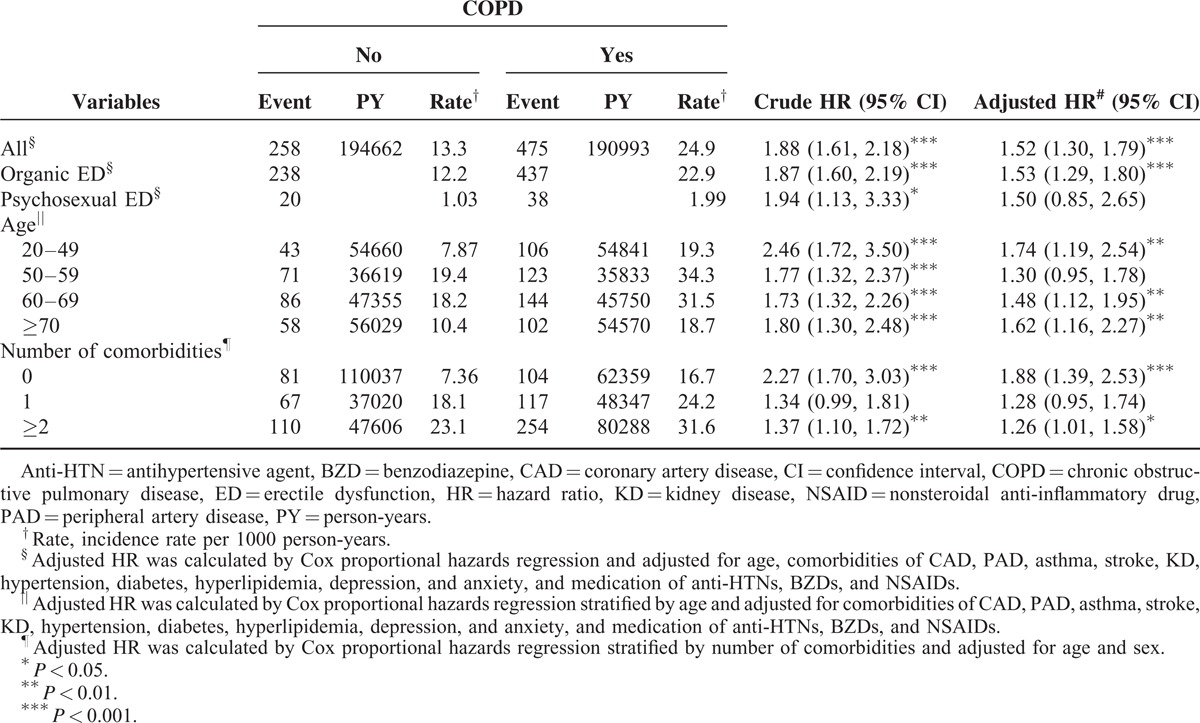
Incidence of Organic and Psychogenic ED and Cox Method Estimated HR of ED for COPD Cohort Compared With Control Cohort by Demographic Characteristics and Comorbidity

The age-specific showed that the ED incidence was the highest for 50- to 59-year-old men in both cohorts. The COPD cohort to non-COPD cohort adjusted HR of ED was not significant for this age group, and was the lowest among age groups. The incidence of ED increased with the number of comorbidities in both cohorts. COPD, with or without comorbidity, was associated with a significantly higher risk of ED than non-COPD.

Table 3 shows not strong increase for the joint effect of developing ED between COPD and comorbidities. Depression had a stronger association with ED than COPD had. Lowered ED HR was observed for those with comorbid stroke. The association between ED and the annual number of emergency room visits and admissions for COPD is shown in Table 4. Compared with non-COPD patients, the HR increased with the number of these services for COPD from 1.51 (95% CI 1.29–1.77) to 5.46 (95% CI 3.03–9.84) and from 1.50 (95% CI 1.28–1.76) to 11.5 (95% CI 5.83–22.6), respectively.

**TABLE 3:**
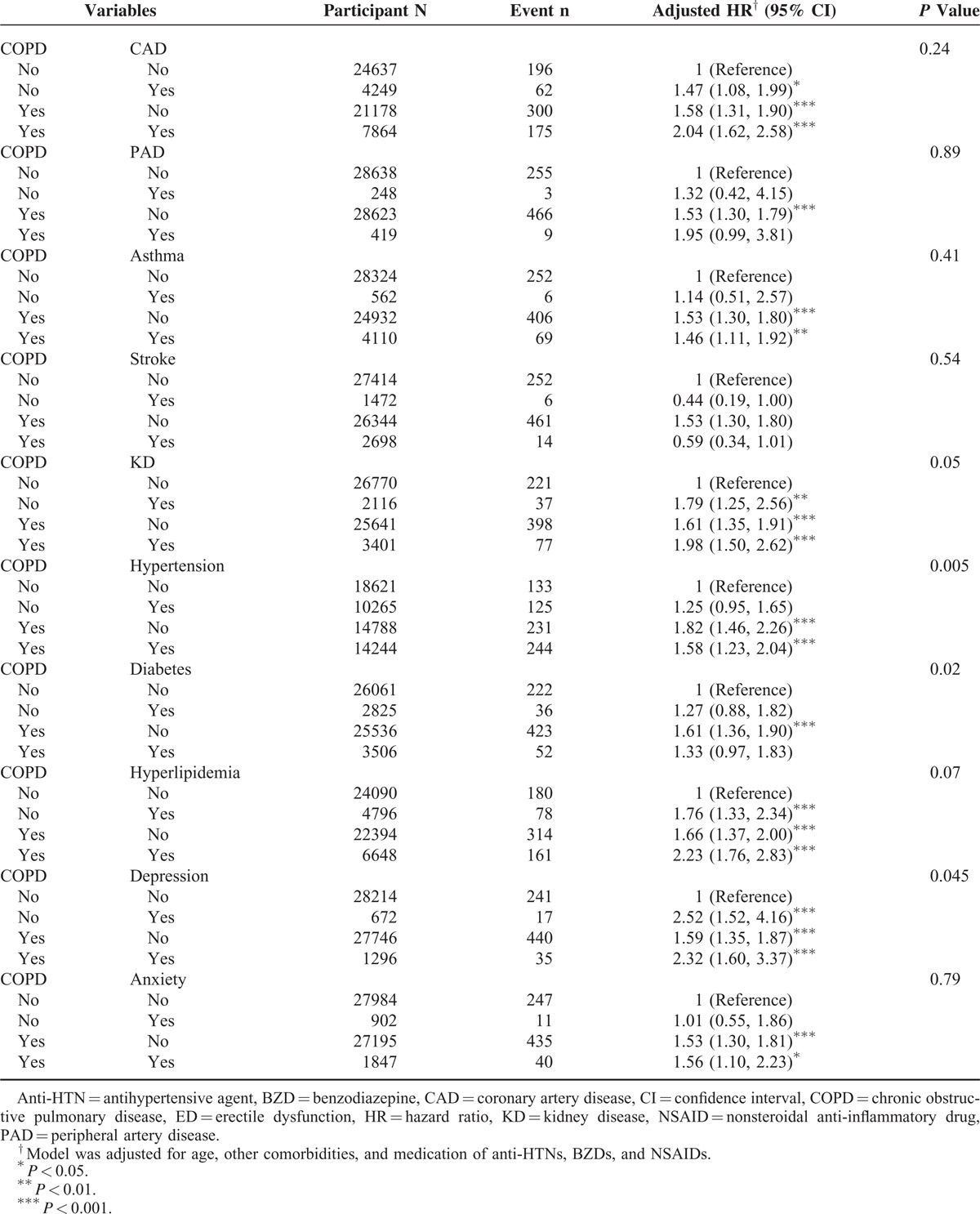
Cox Proportional Hazard Regression Analysis for the Risk of Organic and Psychogenic ED Associated COPD With Joint Effect of Comorbidity

**TABLE 4:**
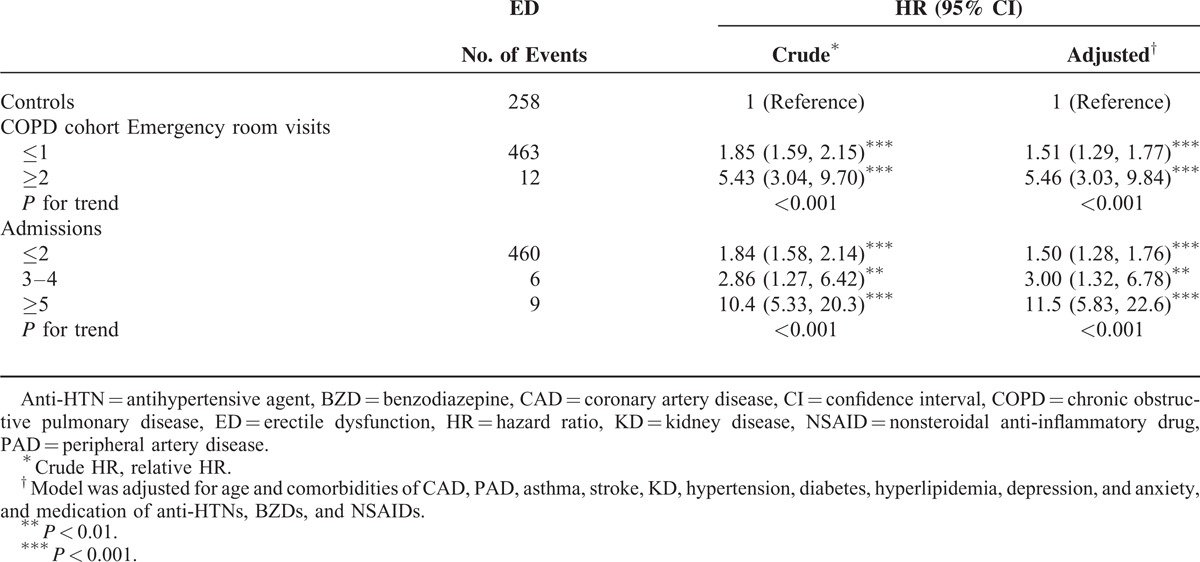
HR of ED Associated With the Number of Annual Emergency Room Visits and Admissions for Patients With COPD

## DISCUSSION

To the best of our knowledge, this is the first nationwide population-based study evaluating the relationship between COPD and subsequent ED risk. A significant hazard of ED was identified among patients with COPD compared with the general population (adjusted HR 1.52, 95% CI 1.29–1.80). Meanwhile, those with more comorbidities were found to have an increased incidence of developing ED in both cohorts. In addition, this study showed that the risk of ED increased as COPD patients required more emergency room visits and admissions. These results support the notion that poor control of COPD status is a key factor affecting ED development.

The definitive mechanism of ED development in COPD patients remains unknown. First, hormonal imbalances should be considered. Androgen deficiency can induce depression, anxiety, anger, fatigue, and sleep disorders. Most important, hormonal imbalance decreases libido, ED, and reduces ejaculation output volume and speed.^11^ Hypogonadism and lower testosterone levels have been reported in males with COPD.^9,12,13^ Therefore, a link between gonadal status and sexual dysfunction in men with COPD is likely. Second, endothelial dysfunction and vascular insufficiency may play an important role. As is well known, cardiovascular disease is the major comorbidity of COPD, and is probably the most frequent and severe condition coexisting with COPD.^14^ In aged patients, ED is most often due to organic causes, the most common factor being atherosclerosis.^15,16^ Not surprisingly, COPD has thus been associated with ED development. Third, respiratory or general symptoms of COPD could also contribute to sexual dysfunction via somatophysical effects; dyspnea, cough, muscular weakness, and the associated reduction of physical activity can directly influence sexual activity in COPD patients.^7,17^ In addition, chronic hypoxia is an important risk associated with pathological conditions, including ED. Recent trials have shown that inadequate oxygen supply impairs nitric oxide synthesis, which subsequently reduces the functional integrity of penile smooth muscles.^18,19^ It has been suggested that ED in COPD may be due to persistent exposure to a hypoxic environment.^20^

In some studies, researchers found a correlation between COPD severity and ED risk.^5,6,9^ In previous versions of the Global Initiative for Chronic Obstructive Lung Disease report, COPD treatment recommendations were based on spirometry only. However, the pulmonary function test is a poor descriptor of disease, and thus, the treatment strategy for COPD should also consider the patient's symptoms and further risk of exacerbations, and therefore, exacerbation history is considered in the evaluation of disease status in COPD.^21^ In the present study, the number of emergency room visits and admissions per year for COPD has been considered to reflect, at least in part, the COPD control status. The results related to emergency room visits and admissions also supported the hypothesis that poor control of COPD status results in an increased risk of ED development.

With regards to relative risk, Karadag et al^7^ reported a 1.05 HR of overall ED between moderate-to-severe COPD and controls, and a 2.85 HR of moderate and severe ED between moderate-to-severe COPD and controls. Kahraman et al^9^ reported a 1.40 HR of overall ED between the COPD group and controls. Thus, the results were compatible to those presented herein (HR 2.17, 95% CI 1.84–2.56). With regards to ED prevalence, previous studies reported a 30% to 79% prevalence in overall COPD patients and a 53% to 87% prevalence in moderate-to-severe COPD patients.^5–9^ The incidence reported herein was far less, probably due to the present study reflecting a relative “real world” scenario wherein the diagnosis of ED is due to a real medical consultation. Thus, the ED patients included were believed to have greater disease severity. In addition, the participants in the previous studies passively received and answered a questionnaire. Not surprisingly, the prevalence of ED in the control group has been reported to be as high as 83%.^7^ Nevertheless, we consider that ED is indeed a much underestimated problem in COPD patients. Clinical physicians should pay more attention to this group of individuals and provide appropriate support.

A major strength of the present study is its use of population-based data that are highly representative of the general population. However, the study has several limitations. First, the diagnoses selected from the ICD-9 code depend on the performance of clinical physicians; we were unable to check their validity. However, the NHI system of Taiwan has been used for various studies over several years.^22–24^ Second, the NHIRD does not contain detailed information regarding smoking habits, body mass index, diet preference, drug use, and family history of systemic diseases, all of which may be associated risk factors for COPD development. In addition, several relevant clinical variables, such as laboratory data, imaging results, culture reports, and pathology findings, were unavailable for the patients. Physicians may not report some clinical conditions in the medical claims without inquiring of patients, such as sexual life after stroke. Stroke is a well-known risk impairing sexual function.^25^ Our data shows that stroke is a protective factor of ED. It is likely that the ED conditions are underreported in our study population. The prevalence of stroke at the baseline was 1.8-fold higher in the COPD cohort than in the non-COPD cohort. Therefore, the protective association of stroke for COPD cohort in this study is misleading.

## CONCLUSION

Patients with COPD are at a significantly higher risk of developing ED compared with the general population regardless of age and presence of comorbidity. The results also support that poor control of COPD status is a key factor affecting ED development.
